# Targeted editing of H3K27me3 reveals its significance in the photoperiodic control of *FLOWERING LOCUS T*

**DOI:** 10.1093/plphys/kiaf470

**Published:** 2025-10-22

**Authors:** Jingyun Lu, Jie Pan, Xiaoyi Li, Huairen Zhang, Ruitian Song, Mande Xue, Jie Li, Qian Liu, Danhua Jiang

**Affiliations:** College of Horticulture, Northwest A&F University, Yangling, Shanxi 712100, China; State Key Laboratory of Seed Innovation, Institute of Genetics and Developmental Biology, Chinese Academy of Sciences, Beijing 100101, China; State Key Laboratory of Seed Innovation, Institute of Genetics and Developmental Biology, Chinese Academy of Sciences, Beijing 100101, China; College of Advanced Agricultural Sciences, University of Chinese Academy of Sciences, Beijing 100039, China; State Key Laboratory of Seed Innovation, Institute of Genetics and Developmental Biology, Chinese Academy of Sciences, Beijing 100101, China; College of Advanced Agricultural Sciences, University of Chinese Academy of Sciences, Beijing 100039, China; Temasek Life Sciences Laboratory, National University of Singapore, Singapore 117604, Singapore; State Key Laboratory of Seed Innovation, Institute of Genetics and Developmental Biology, Chinese Academy of Sciences, Beijing 100101, China; State Key Laboratory of Seed Innovation, Institute of Genetics and Developmental Biology, Chinese Academy of Sciences, Beijing 100101, China; College of Advanced Agricultural Sciences, University of Chinese Academy of Sciences, Beijing 100039, China; State Key Laboratory of Seed Innovation, Institute of Genetics and Developmental Biology, Chinese Academy of Sciences, Beijing 100101, China; College of Advanced Agricultural Sciences, University of Chinese Academy of Sciences, Beijing 100039, China; State Key Laboratory of Seed Innovation, Institute of Genetics and Developmental Biology, Chinese Academy of Sciences, Beijing 100101, China; College of Advanced Agricultural Sciences, University of Chinese Academy of Sciences, Beijing 100039, China; Temasek Life Sciences Laboratory, National University of Singapore, Singapore 117604, Singapore; State Key Laboratory of Seed Innovation, Institute of Genetics and Developmental Biology, Chinese Academy of Sciences, Beijing 100101, China; College of Advanced Agricultural Sciences, University of Chinese Academy of Sciences, Beijing 100039, China; Temasek Life Sciences Laboratory, National University of Singapore, Singapore 117604, Singapore

## Abstract

The repressive histone modification H3K27me3 plays a causal role in determining gene expression patterns.

Dear Editor,

Epigenetic modifications play pivotal roles in controlling the function of eukaryotic genome. It is suggested that one of the major functions of epigenetic modification is to regulate transcriptional activity. However, evaluating the direct impact of an epigenetic modification on individual genes can be challenging, as the traditional analysis with mutants of epigenetic modifiers (epi-modifier) such as “writers” and “erasers” may reflect widespread and indirect changes, making it difficult to establish causality between altered epigenetic modifications and gene expression. In addition, some epi-modifiers may have additional functions independent of their enzymatic activities (Morgan and Shilatifard 2023).

The timing of the floral transition, or flowering, is crucial for plant reproductive success and crop yield. Many plants align their flowering time with changes in day length, or photoperiod, which is measured by leaves. The inductive photoperiodic condition triggers the production of FLOWERING LOCUS T (FT) in leaves, and it is then transported into the shoot apex to induce flowering (Corbesier et al. 2007; Turck et al. 2008; Adrian et al. 2010). In the long day (LD) plant *Arabidopsis*, the LD condition promotes *FT* transcription, especially at the end of LD, while *FT* is expressed at very low levels under short day (SD) conditions (Kobayashi et al. 1999). The *FT* locus is marked by the repressive H3K27 trimethylation (H3K27me3) (Jiang et al. 2008), a histone modification that is considered to be critical in programming development and environmental responses. Although several studies have suggested the importance of H3K27me3 in *FT* regulation (Jiang et al. 2008; Wang et al. 2014), whether it directly impacts on *FT* transcription and its photoperiodic control remains elusive.

Here, we focused on the *FT* locus and analyzed the significance of H3K27me3 on its transcriptional regulation by directly and specifically reducing its accumulation levels at *FT* with a clustered regularly interspaced short palindromic repeats (CRISPR)/catalytically dead Cas9 (dCas9)-based targeting system. We adapted the CRISPR/dCas9 SunTag system that was successfully used for modifying DNA methylation in plants (Gallego-Bartolome et al. 2018; Papikian et al. 2019; Tang et al. 2022). This system includes 3 modules: guide RNA(s), dCas9 fused with SunTag (GCN4 peptide repeats), and epi-modifier (effector) tagged with a single-chain variable fragment (scFv) antibody and a superfolder GFP (sfGFP) (Fig. 1A). scFv binds to GCN4, and therefore multiple copies of epi-modifier (effector) are recruited to specific loci by CRISPR-dCas9 (Fig. 1B). Each module may vary depending on specific applications. For instance, guide RNA(s) and epi-modifiers would differ for different genomic targets and epigenetic modifications that are to be edited, respectively. In addition, different promoters can be utilized to drive the expression of these modules in a spatial–temporal manner. To facilitate the interchangeability of these variable elements, we employed a GreenGate cloning system and divided these 3 modules into 6 cassettes, which can be rapidly and efficiently assembled in a single reaction (Fig. 1, C and D and Supplementary materials and methods) (Lampropoulos et al. 2013). Thereby, different applications only require the changing of a few building blocks. In this study, we have cloned several cassettes that are readily available for use (Supplementary Table S1).

**Figure 1.**
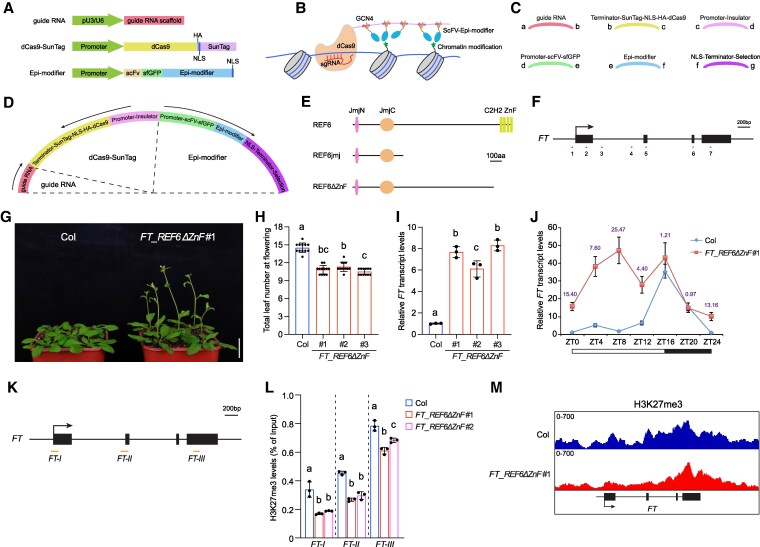
Targeted editing of H3K27me3 at *FT* alters its expression pattern and the timing of flowering. **A)** Schematic representation of the 3 modules in the CRISPR/dCas9 SunTag. NLS, nuclear localization signal; dCas9, catalytically dead Cas9; HA, hemagglutinin tag; SunTag, GCN4 peptide repeats; scFV, single-chain variable fragment; sfGFP, superfolder GFP. **B)** Schematic depicting of dCas9–SunTag–epi-modifier on editing chromatin modifications. **C)** Six cassettes in the GreenGate cloning system. **D)** The final assembly of the 3 modules that form dCas9–SunTag–epi-modifier in a binary vector. **E)** Diagram of REF6, REF6jmj, and REF6ΔZnF used in this study. **F)** Schematic representation of the positions of guide RNAs g1–g7 at the *FT* locus. Arrow indicates transcription start site, filled boxes indicate exons, and numbers indicate guide RNA targeted sites. **G)** The flowering phenotypes of Col and *FT_REF6ΔZnF* #1 grown in LD. Scale bar, 2 cm. **H)** The flowering time of Col, *FT_REF6ΔZnF* #1, *FT_REF6ΔZnF* #2, and *FT_REF6ΔZnF* #3 grown in LD. The total number of primary rosette and cauline leaves at flowering were counted; 12 plants were scored for each line. Values are means ± Sd. Significance of differences was tested using 1-way ANOVA with Tukey's test (*P* < 0.05), with different letters indicating statistically significant differences. **I)** Relative *FT* transcript levels in Col, *FT_REF6ΔZnF* #1, *FT_REF6ΔZnF* #2, and *FT_REF6ΔZnF* #3 at zeitgeber time (ZT) 4 under LD determined by RT-qPCR. *TUBULIN 2* (*TUB2*) was used as an endogenous control. Values are means ± Sd of 3 biological replicates. Significance of differences was tested using 1-way ANOVA with Tukey's test (*P* < 0.05), with different letters indicating statistically significant differences. **J)**  *FT* transcript levels in Col and *FT_REF6ΔZnF* #1 over a 24-h LD cycle determined by RT-qPCR. *TUB2* was used as an endogenous control. Values are means ± Sd of 3 biological replicates. The *FT* transcript levels in Col at ZT0 are set as 1. Numbers indicate the average relative fold changes at each time point. White and dark bars below the *x* axis mark light and dark periods, respectively. **K)** Primer localization used for ChIP-qPCR at the *FT* gene body. Arrow indicates transcription start site, filled boxes indicate exons, and “*FT-I*”, “*FT-II*”, and “*FT-III*” indicate regions examined by ChIP-qPCR. **L**) H3K27me3 levels at *FT* in Col, *FT_REF6ΔZnF* #1, and *FT_REF6ΔZnF* #2 at ZT4 under LD determined by ChIP-qPCR. Values are means ± Sd of 3 biological replicates. Statistical significance was evaluated using 1-way ANOVA with Tukey's test (*P* < 0.05), with different letters indicating statistically significant differences. **M)** Genome browser view of H3K27me3 ChIP-seq signals in Col and *FT_REF6ΔZnF* #1 encompassing the *FT* locus at ZT4.

In *Arabidopsis*, several H3K27 demethylases have been identified, and among them RELATIVE OF EARLY FLOWERING 6 (REF6)/JUMONJI 12 (JMJ12) is well characterized (Lu et al. 2011; Cui et al. 2016). We first targeted the catalytic JMJ domain of REF6 (REF6jmj) to the *FT* locus (Fig. 1E and Supplementary Fig. S1A). The ubiquitous *Arabidopsis UBIQUITIN 10* (*UBQ10*) promoter was used to drive the expression of dCas9-SunTag and REF6jmj. Because H3K27me3 usually accumulates across the whole gene body, a cluster of guide RNAs g1–g7 covering *FT* were used (Fig. 1F and Supplementary Table S2). Removal of H3K27me3 from *FT* is expected to activate its transcription, leading to accelerated flowering. However, no obvious phenotypes were observed (Supplementary Fig. S1B). It is possible that the REF6 JMJ domain alone may not be sufficient to catalyze H3K27 demethylation in vivo. Therefore, we targeted full-length REF6 to the *FT* locus (Fig. 1E). However, this strategy also failed to accelerate flowering (Supplementary Fig. S1C).

The REF6 C-terminal contains 4 Cys_2_His_2_ zinc fingers (ZnFs), which facilitate REF6 targeting to the genome via recognizing a CTCTGYTY motif (Cui et al. 2016; Pan et al. 2022). We suspected that the scFv-tagged full-length REF6 may preferentially binds to in vivo REF6 targets through its ZnFs, preventing its association with the *FT*-localized dCas9-SunTag. Therefore, at last, we targeted REF6 without its C2H2 ZnFs (REF6ΔZnF) (Fig. 1E). In this case, several transgenic lines (*FT_REF6ΔZnF*) showed early flowering phenotypes and increased *FT* transcript levels (Fig. 1G to I), while targeting the enzymatic activity-mutated REF6ΔZnF (dREF6ΔZnF) to *FT* or only expressing dCas9-SunTag and REF6ΔZnF without guide RNAs had no effect on flowering and *FT* expression (Supplementary Fig. S1, A and D to F). Interestingly, tethering REF6ΔZnF to the *FT* locus altered the *FT* expression rhythm under LD, with its transcript levels increasing mainly during the noninductive period (Fig. 1J). Although dCas9 was successfully targeted to the *FT* locus when coexpressed with guide RNAs and REF6, REF6jmj, REF6ΔZnF, or dREF6ΔZnF, a reduction in H3K27me3 at *FT* was observed only upon expression of REF6ΔZnF (Fig. 1K to M and Supplementary Fig. S2A to C), consistent with changes in flowering time and *FT* expression. In addition, genome-wide H3K27me3 levels were comparable to those in wild type (WT) Columbia (Col) (Supplementary Fig. S2, D and E), including the guide RNA-untargeted regulatory regions upstream and downstream of *FT* (Supplementary Fig. S2, F and G) (Adrian et al. 2010; Cao et al. 2014; Zicola et al. 2019; Takagi et al. 2023), highlighting the specificity of REF6ΔZnF targeting. Notably, the reduction in H3K27me3 was more pronounced toward the 5′ half of the *FT* locus, where H3K27me3 is moderately enriched (Fig. 1K to M). This may reflect the higher density of guide RNAs targeting this region, leading to stronger recruitment of REF6ΔZnF (Fig. 1F and Supplementary Fig. S2B), or alternatively, the strong accumulation of H3K27me3 at the 3′ half of the *FT* locus may somehow impede targeted H3K27me3 removal from this region.

Although decreasing H3K27me3 abolished the *FT* expression rhythm at LD, flowering was only mildly accelerated. We speculated that by decreasing H3K27me3 at the *FT* locus, the induction of its expression at the normally noninductive period may not generate a strong impact on flowering at LD, which can induce the *FT* expression at dusk in WT anyway (Fig. 1J). Thus, the flowering time of H3K27me3-manipulated plants were further examined at SD. *FT_REF6ΔZnF* lines flowered much earlier than WT under the SD conditions (Fig. 2, A and B). Compared with LD, SD is noninductive to the *FT* transcription in *Arabidopsis* (Turck et al. 2008). At SD, *FT* is constantly expressed at low levels, with only a slight increase during daytime (Supplementary Fig. S3). However, targeting REF6ΔZnF to decrease H3K27me3 strongly promoted the *FT* transcript levels at SD (Fig. 2, C and D).

**Figure 2.**
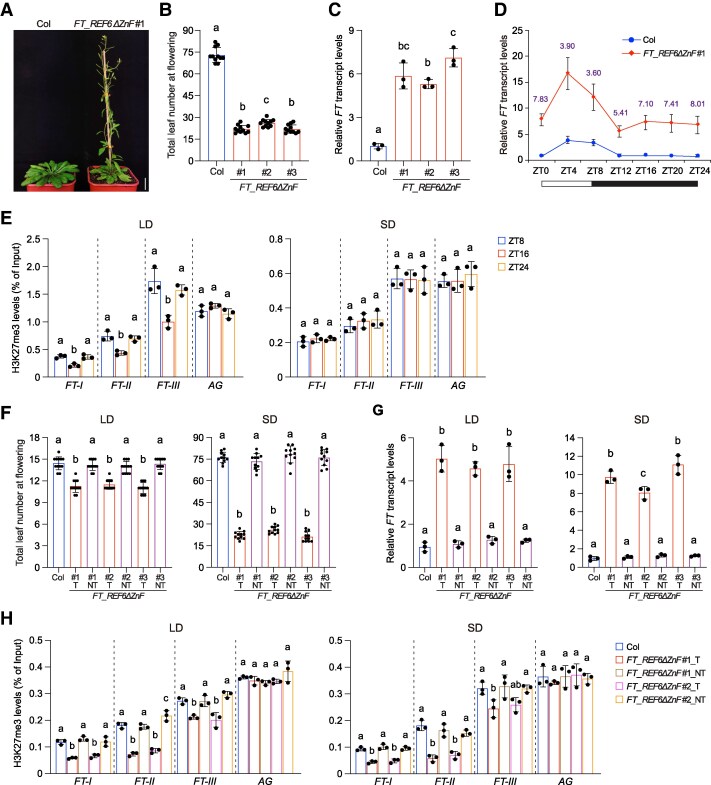
H3K27me3 accumulates to repress *FT* transcription during the noninductive period. **A)** The flowering phenotypes of Col and *FT_REF6ΔZnF* #1 grown in SD. Scale bar, 2 cm. **B)** The flowering time of Col, *FT_REF6ΔZnF* #1, *FT_REF6ΔZnF* #2, and *FT_REF6ΔZnF* #3 grown in SD. The total number of primary rosette and cauline leaves at flowering were counted; 11 plants were scored for each line. Values are means ± Sd. Significance of differences was tested using 1-way ANOVA with Tukey's test (*P* < 0.05), with different letters indicating statistically significant differences. **C)** Relative *FT* transcript levels in Col, *FT_REF6ΔZnF* #1, *FT_REF6ΔZnF* #2, and *FT_REF6ΔZnF* #3 at ZT4 under SD determined by RT-qPCR. *TUB2* was used as an endogenous control. Values are means ± Sd of 3 biological replicates. Significance of differences was tested using 1-way ANOVA with Tukey's test (*P* < 0.05), with different letters indicating statistically significant differences. **D)**  *FT* transcript levels in indicated lines over a 24-h SD cycle determined by RT-qPCR. *TUB2* was used as an endogenous control. Values are means ± Sd of 3 biological replicates. The *FT* transcript levels in Col at zeitgeber time (ZT) 0 are set as 1. Numbers indicate the average relative fold changes at each time point. Bars below the *x* axis indicate light and dark periods. **E)** H3K27me3 levels at *FT* under LD and SD in WT Col determined by ChIP-qPCR. *AGAMOUS* (*AG*), a H3K27me3-enriched locus, is used as a control. Values are means ± Sd of 3 biological replicates. Statistical significance was evaluated using 1-way ANOVA with Tukey's test (*P* < 0.05), with different letters indicating statistically significant differences. **F)** The flowering time of T3 lines grown in LD and SD. The total number of primary rosette and cauline leaves at flowering were counted; 11 plants were scored for each line. Values are means ± Sd. T, T3 plants with the transgene; NT, T3 plants without the transgene. Statistical significance was evaluated using 1-way ANOVA with Tukey's test (*P* < 0.05), with different letters indicating statistically significant differences. **G)** Relative *FT* transcript levels in T3 lines at ZT4 under LD and SD. Rosette leaves of plants with or without the transgene were collected for RNA extraction. *TUB2* was used as an endogenous control. Values are means ± Sd of 3 biological replicates. Significance of differences was tested using 1-way ANOVA with Tukey's test (*P* < 0.05), with different letters indicating statistically significant differences. **H)** H3K27me3 levels at *FT* in T3 lines at ZT4 under LD and SD determined by ChIP-qPCR. Rosette leaves of plants with or without the transgene were collected for chromatin extraction. Values are means ± Sd of 3 biological replicates. Statistical significance was evaluated using 1-way ANOVA with Tukey's test (*P* < 0.05), with different letters indicating statistically significant differences.

The above results obtained at LD and SD demonstrate that decreasing H3K27me3 at *FT* by targeting REF6ΔZnF predominantly induces its transcription under the noninductive conditions. Previous studies have shown that disrupting Polycomb repressive complex 2 (PRC2) or PRC1 components, which reduces H3K27me3 levels at *FT*, strongly derepresses *FT* transcription at the noninductive times of LD, and the accumulation of H3K27me3 at *FT* oscillates, with higher levels at the noninductive period (Wang et al. 2014). We thus sought to investigate the temporal distribution patterns of H3K27me3 at *FT* under both LD and SD using ChIP-qPCR. As previously reported, H3K27me3 levels were reduced at dusk under LD (Fig. 2E) (Wang et al. 2014), coinciding with the induction of *FT* transcription. However, H3K27me3 did not show fluctuations under SD (Fig. 2E). These results suggest that H3K27me3 primarily represses *FT* transcription under noninductive conditions.

*FT* expression is confined to phloem companion cells (Chen et al. 2018). We further expressed REF6ΔZnF with the *SUCROSE TRANSPORTER2* (*SUC2*) promoter (Supplementary Fig. S4A), which is specifically active in the phloem companion cells (Stadler and Sauer 1996). This led to both early flowering and increased *FT* transcription (Supplementary Fig. S4, B and C), suggesting that reduction of H3K27me3 in companion cells is sufficient to activate *FT* expression. Finally, we examined whether the early flowering phenotype observed in the *FT_REF6ΔZnF* lines is heritable after the transgene is segregated away. However, T3 plants without the transgene exhibited flowering time, *FT* transcript levels, and H3K27me3 levels at *FT* comparable to those of WT (Fig. 2F to H). This suggests that *FT* activation depends on the presence of a low H3K27me3 state at the *FT* locus and that this epigenetic state is not stably inherited across generations in the absence of the transgene. Taken together, our findings provide direct evidence for the causal importance of H3K27me3 in repressing *FT* transcription and highlight the potential for fine-tuning gene expression patterns and phenotypic traits in plants by editing epigenetic modifications.

## Supplementary Material

kiaf470_Supplementary_Data

## Data Availability

The ChIP-seq data generated in this study are available in the GEO repository under the accession number GSE303376 (https://www.ncbi.nlm.nih.gov/geo/query/acc.cgi?acc=GSE303376).

## Supplementary data

The following materials are available in the online version of this article.

**Supplementary Figure S1**. REF6-related constructs and analyses.

**Supplementary Figure S2**. Analysis of dCas9 occupancy and H3K27me3 enrichment.

**Supplementary Figure S3**. *FT* transcript levels in WT Col over a 24-h LD or SD cycle determined by RT-qPCR.

**Supplementary Figure S4**. Targeted editing of H3K27me3 at *FT* in phloem companion cells accelerates flowering.

**Supplementary Table S1**. Constructed cassettes for the cloning of epigenetic editing constructs.

**Supplementary Table S2**. Guide RNA sequences used in this study.

**Supplementary Table S3**. Primers used in this study.

**Supplementary Table S4**. Aligned results and correlation analysis in ChIP-seq experiments.
