# Supplementary material for: Targeted editing of H3K27me3 reveals its significance in the photoperiodic control of FLOWERING LOCUS T
Source: Plant Physiol. 2025 Oct 22;199(2):kiaf470. doi: 10.1093/plphys/kiaf470 (PMC12541365; doi:10.1093/plphys/kiaf470)
Supplement: kiaf470_Supplementary_Data [file kiaf470_supplementary_data.pdf]

Supplementary materials and methods

Plant materials and growth conditions

All plant materials used in this study are in the Columbia (Col) background. Plants were grown in the growth room under long-day conditions (LD) (21°C,16h light/8h dark) or short-day conditions (SD) (21°C, 8h light/16h dark) on soil or 1/2 MS plates. Flowering time was measured by counting the number of primary rosette and cauline leaves produced at the time of bolting.

Plasmid construction

The GreenGate cloning system was adapted from Lampropoulos et al., 2013. To clone the guide RNA expression cassette (a-b), sequences of AtU3/U6 promoters were PCR-amplified from Addgene plasmids #66198, #66200, #66202, #66203 (Ma et al., 2015), while guide RNA sequences are listed in Supplementary Table S2. For the cloning of the dCas9-SunTag (b-c), the NLS-dCas9-HA-SunTag-OCS terminator sequences were amplified from Addgene plasmid #106435 (Gallego-Bartolome et al., 2018). To clone the insulator and the promoter that drives the expression of dCas9-SunTag (c-d), the Insulator-pAtUBQ10 sequences were amplified from Addgene plasmid #106435 (Gallego-Bartolome et al., 2018), and the pZmUBI promoter sequences were amplified from Addgene plasmids #66187 (Ma et al., 2015) and then fused with insulator. For the cloning of Promoter-scFV-sfGFP (d-e), the pAtUBQ10-scFV-sfGFP sequences were amplified from Addgene plasmid #106435 (Gallego-Bartolome et al., 2018), the pAtSUC2 promoter sequences were amplified from the *Arabidopsis* genomic DNA and then fused with scFV-sfGFP, and the pZmUBI promoter sequences were amplified from Addgene plasmids #66187 (Ma et al., 2015) and then fused with scFV-sfGFP. For the cloning of epi-modifiers (e-f), their sequences were amplified from cDNA generated from WT Col. To generate enzymatically inactive REF6△ZnF (dREF6△ZnF), the Histidine (H) 246 residue in REF6 was mutated to A (Lu et al., 2011). To clone the NLS-NOS terminator-Hygromycin selection mark (f-g), their sequences were amplified from Addgene plasmid #106435 (Gallego-Bartolome et al., 2018). These six elements were finally assembled in a binary vector pZ304. All plasmids listed in Supplementary Table S1 are deposited to Addgene.

RT-qPCR

To analyze the transcript levels of *FT*, total RNA was extracted either from 10-day-old seedlings grown under LD or SD, or from rosette leaves of plants with or without the transgene, using the Eastep Super total RNA Extraction Kit (Promega, LS1040). Reverse transcription was performed using the HiScript III 1st Strand cDNA Synthesis Kit (Vazyme, R312-02). Real-time qPCR was performed on an Applied Biosystems QuantStudio 6 Flex Real-Time PCR System or a Bio-Rad CFX384 Touch Real-Time PCR Detection System using ChamQ Universal SYBR qPCR Master Mix (Vazyme, Q711-02). *TUBULIN 2* (*TUB2*) was used as an endogenous control for normalization. Three or four independent biological replicates were performed for each line and condition. Primers used for amplification are listed in Supplementary Table S3.

ChIP-seq

Ten-day-old seedlings materials grown under LD were collected at ZT4 and fixed with 1% formaldehyde. After nuclei extraction, chromatin was digested by micrococcal nuclease digestion. Immunoprecipitation was performed with an anti-H3K27me3 (Millipore, 07-449) antibody. Two independent biological replicates were performed. The antibody-recovered DNA were used for library preparation with a VAHTS Universal DNA library prep kit for Illumina (Vazyme, ND607) according to the manufacturer’s instructions. Prepared libraries were sequenced on the Hi-Seq X Ten platform, and paired-end 150-bp reads were generated.

Adapter trimming was performed and low quality reads were filtered with fastp version 0.20.1. Reads were mapped to the *Arabidopsis* genome (TAIR10) with Botiew2 version 2.4.2, and filtered for duplicated reads using Picard version 2.24.0 MarkDuplicates (https://github.com/broadinstitute/picard) (Supplementary Table S4). For data visualization, data from two biological replicates were merged and bigwig coverage files were generated using deepTools utility bamCoverage with a bin size of 10bp. Average ChIP-seq profiles were plotted using deepTools utility plotProfile.

ChIP-qPCR

ChIP experiments were performed using sonicated chromatin extracted either from 10-days-old seedlings grown under LD or SD, or from rosette leaves of plants with or without the transgene. Immunoprecipitations were performed with an anti-H3K27me3 (Millipore 07-449) or anti-HA (CST 3724) antibody. The amount of immunoprecipitated

DNA was quantified by real-time PCR. Three independent biological replicates were performed for each line and condition. Primers used for amplification are specified in Supplementary Table S3.

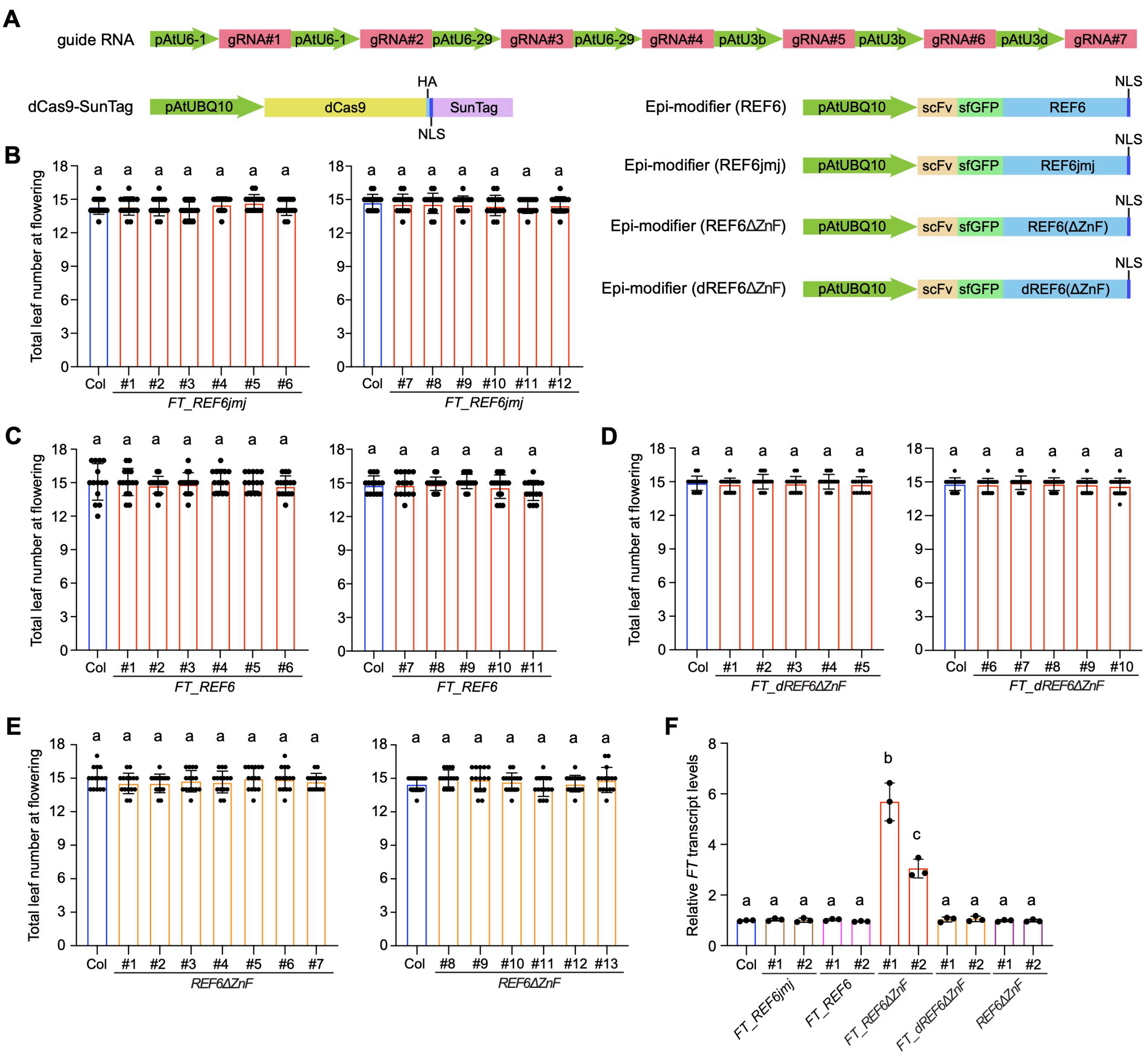

Supplementary Figure S1. REF6-related constructs and analyses

A. Schematic representation of the three modules in the *FT_REF6*, *FT_REF6jmj*, *FT_REF6ΔZnF*, or *FT_dREF6ΔZnF* construct. NLS: nuclear localization signal; dCas9: nuclease-inactive Cas9; HA: Hemagglutinin tag; SunTag: GCN4 peptide repeats; scFV: single-chain variable fragment; sfGFP: superfolder GFP.

B-D. The flowering time of indicated transgenic lines in LD. The total number of primary rosette and cauline leaves at flowering were counted; 14-15 plants were scored for each line. Values are means ± sd. Significance of differences was tested using one-way ANOVA with Tukey's test ($P < 0.05$), with different letters indicating statistically significant differences.

E. The flowering time of transgenic lines expressing dCas9-SunTag and REF6ΔZnF without guide RNAs in LD. The total number of primary rosette and cauline leaves at flowering were counted; 15 plants were scored for each line. Values are means ± sd.

Significance of differences was tested using one-way ANOVA with Tukey's test ($P < 0.05$), with different letters indicating statistically significant differences.

F. Relative *FT* transcript levels in Col, *FT_REF6jmj*, *FT_REF6*, *FT_REF6ΔZnF*, *FT_dREF6ΔZnF*, and *REF6ΔZnF* at ZT4 under LD determined by RT-qPCR. Two lines were selected for each construct. *TUB2* was used as an endogenous control. Values are means ± sd of three biological replicates. Significance of differences was tested using one-way ANOVA with Tukey's test ($P < 0.05$), with different letters indicating statistically significant differences.

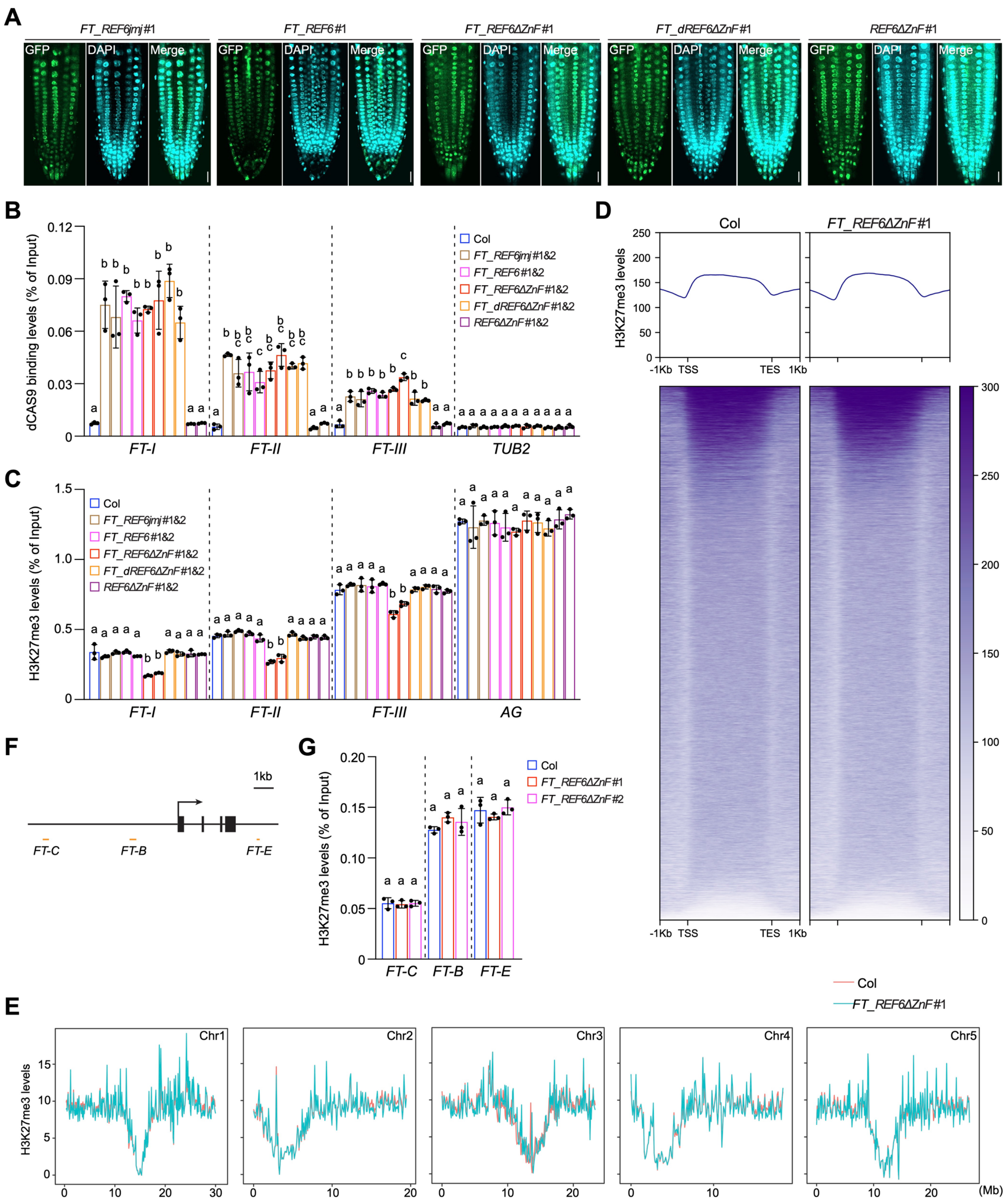

Supplementary Figure S2. Analysis of dCas9 occupancy and H3K27me3 enrichment

A. GFP signals in root cells of transgenic lines. Nuclei were counterstained with 4',6-diamidino-2-phenylindole (DAPI). Scale bars, 10 μm.

B. dCas9 binding levels at *FT* in transgenic lines at ZT4 under LD determined by ChIP-qPCR. *TUB2* is used as a control. Values are means ± sd of three biological replicates.

Statistical significance was evaluated using one-way ANOVA with Tukey's test ($P < 0.05$), with different letters indicating statistically significant differences.

C. H3K27me3 levels at *FT* in transgenic lines at ZT4 under LD determined by ChIP-qPCR. *AGAMOUS* (*AG*), a H3K27me3-enriched locus, is used as a control. Values are means ± sd of three biological replicates. Statistical significance was evaluated using one-way ANOVA with Tukey's test ($P < 0.05$), with different letters indicating statistically significant differences.

D. Metaplot and heatmap of H3K27me3 ChIP-seq signals in Col and *FT_REF6ΔZnF* #1 over all genes. TSS: transcription start site; TES: transcription end site.

E. H3K27me3 ChIP-seq signals over *Arabidopsis* chromosomes in Col and *FT_REF6ΔZnF* #1. Signals were calculated in 100kb bins.

F. Primer localization used for ChIP-qPCR at *FT* regulatory regions. Arrow indicates transcription start site, filled boxes indicate exons, orange lines indicate regions examined by ChIP-qPCR.

G. H3K27me3 levels at *FT* regulatory regions in Col, *FT_REF6ΔZnF* #1, and *FT_REF6ΔZnF* #2 at ZT4 under LD determined by ChIP-qPCR. Values are means ± sd of three biological replicates. Statistical significance was evaluated using one-way ANOVA with Tukey's test ($P < 0.05$), with different letters indicating statistically significant differences.

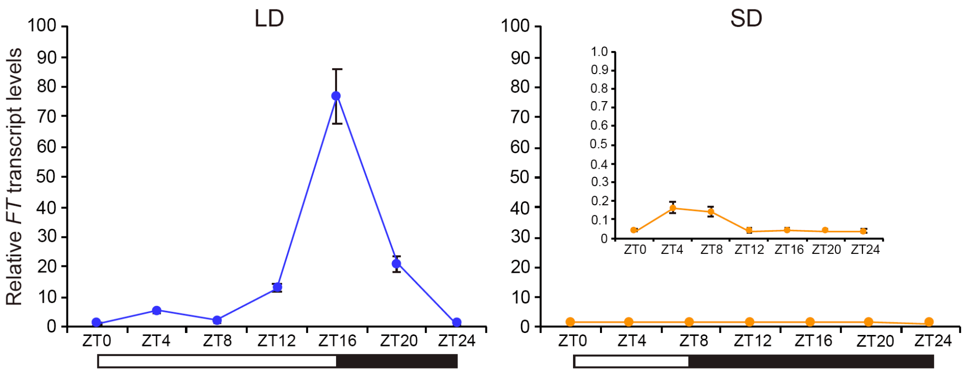

Supplementary Figure S3. *FT* transcript levels in WT Col over a 24-h LD or SD cycle determined by RT-qPCR

TUB2 was used as an endogenous control. Values are means ± sd of three biological replicates. The *FT* transcript levels in Col at ZT0 under LD is set as 1. Numbers indicate the average relative fold changes at each time point. White and dark bars that below the x-axis mark light and dark periods, respectively. The same data in SD is presented using two different y-axis ranges (0-100 and 0-1).

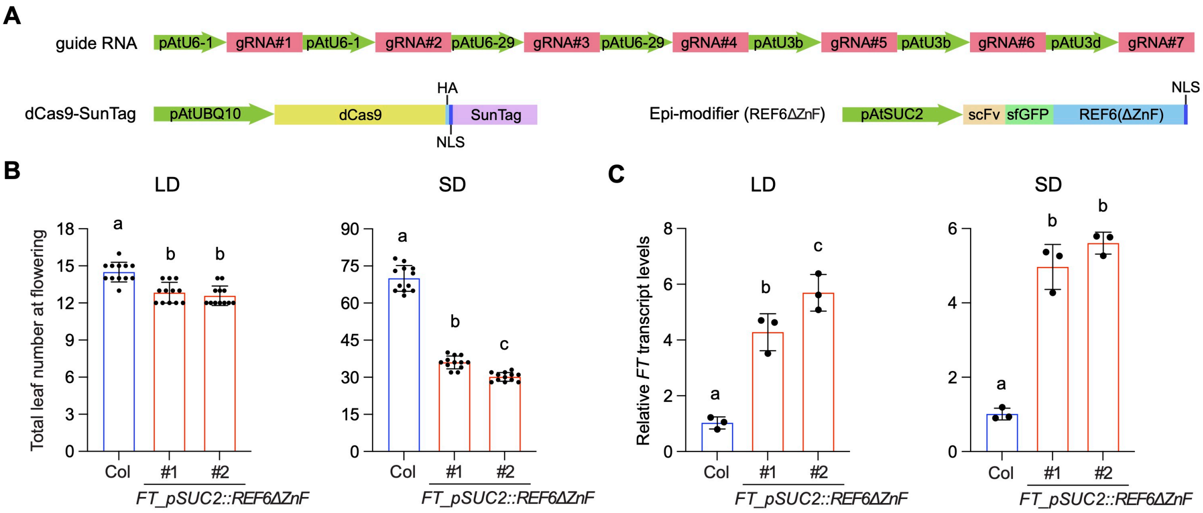

Supplementary Figure S4. Targeted editing of H3K27me3 at *FT* in phloem companion cells accelerates flowering

A. Schematic representation of the three modules in the *FT_pSUC2::REF6ΔZnF* construct.

B. The flowering time of *FT_pSUC2::REF6ΔZnF* transgenic lines grown in LD and SD. The total number of primary rosette and cauline leaves at flowering were counted; 12 plants were scored for each line. Values are means ± sd. Significance of differences was tested using one-way ANOVA with Tukey's test ($P < 0.05$), with different letters indicating statistically significant differences.

C. Relative *FT* transcript levels in *FT_pSUC2::REF6ΔZnF* transgenic lines at ZT4 under LD and SD determined by RT-qPCR. *TUB2* was used as an endogenous control. Values are means ± sd of three biological replicates. Significance of differences was tested using one-way ANOVA with Tukey's test ($P < 0.05$), with different letters indicating statistically significant differences.

Supplementary Table S1. Constructed cassettes for the cloning of epigenetic editing constructs

Overhangs	Type	Available cassettes
a-b	guide RNA	*FT*_g1-7
b-c	Terminator-SunTag-HA-dCas9-NLS	OCS terminator-SunTag-HA-dCas9-NLS
c-d	Promoter-Insulator	AtUBQ10 promoter-Insulator
		ZmUBI Promoter-Insulator
d-e	Promoter-scFV-sfGFP	AtUBQ10 Promoter-scFV-sfGFP
		AtSUC2 Promoter-scFV-sfGFP
		ZmUBI Promoter-scFV-sfGFP
e-f	Epi-modifier	AtATX1set
		AtJMJ14
		AtLDL1
		AtCLF
		AtCLFset
		AtFIE
		AtVAL1
		AtREF6
		AtREF6jmj
		AtREF6ΔZnF
		AtHAC1cd
		AtHDA6
		AtATXR5set
		NtDRMcd
		TET1cd
f-g	NLS-Terminator-selection	NLS-NOS terminator-Hygromycin resistance

Supplementary Table S2. Guide RNA sequences used in this study

Gene	Guide RNA	Sequences (5’ to 3’)
FT	*guide RNA1*	GGATTTGCATTAACTCGGGT
	guide RNA2	GGTTACTTATGGCCAAAGAG
	guide RNA3	GCGTGGGGCATTTTTAACCA
	guide RNA4	TATAGCAGAGAGTATCTTAA
	guide RNA5	ACCAATGGAGATATTCTCGG
	guide RNA6	AGGTTGTTCCAGTTGTAGCA
	guide RNA7	CTACAATCTCGGCCTTCCCG

Supplementary Table S3. Primers used in this study

Experiment	Amplified region	Sequences (5' to 3')
RT-qPCR	*FT*	GTCCTAGCAACCCTCACCTC
		CCTGCAGTGGGACTTGGATT
	TUB2	ACTGTCTCCAAGGGTTCCAGG
		AAGAACCATGCACTCATCAGC
ChIP-qPCR	*FT_I*	TAAATATGTGTAGAGGGTTCATGCC
		CTTTGATCTTGAACAAACAGGTGGT
	FT_II	AAGACCTTTTGCTTTCTTGATTTCTTTG
		TGAGGGTTGCTAGGACTTGGAA
	FT_III	TGGCGCCAGAACTTCAACACT
		GCCACTCTCCCTCTGACAATTGTA
	FT_C	TCTGATTTGGGGTTCAAAA
		TCGAACTGATTCCGATTGAA
	FT_B	CACAAGTGGCGGACAATCCATC
		CTACGATCGACCATTGATAATCG
	FT_E	ACATATCCACTCAGAGGAAGGTC
		GTAACCTTCTTGTATGGCTGATG
	AG	ATGCTGAAGTCGCACTCATCGTCT
		GAGCACGAGAAGAAGAAGAAACCTG
	TUB2	ATCCGTGAAGAGTACCCAGAT
		AAGAACCATGCACTCATCAGC

Supplementary Table S4. Aligned results and correlation analysis in ChIP-seq experiments

Samples	Aligned reads	Correlation (R-value)
Col_H3K27me3_ZT4 rep1	15,658,090	0.9671
Col_H3K27me3_ZT4 rep2	19,145,076	
FT_REF6ΔZnF #1_H3K27me3_ZT4 rep1	19,390,518	0.9751
FT_REF6ΔZnF #1_H3K27me3_ZT4 rep2	17,333,173	
